# Role of motility-related protein-1 in promoting the development of several types of cancer (Review)

**DOI:** 10.3892/ol.2014.1786

**Published:** 2014-01-09

**Authors:** HAN XUAN, XIAOTONG HU, JINWEN HUANG

**Affiliations:** 1 Department of Hematology, Sir Run Run Shaw Hospital, Zhejiang University, Hangzhou, Zhejiang 310016, P.R. China; 2 Biomedical Research Center and Key Laboratory of Biotherapy of Zhejiang Province, Sir Run Run Shaw Hospital, Zhejiang University, Hangzhou, Zhejiang 310016, P.R. China

**Keywords:** CD9, cell motility, tumor metastasis, cancer stem cell

## Abstract

Motility-related protein-1 (CD9), a type of cell surface glycoprotein comprising a four-pass transmembrane domain that forms multimeric complexes with other cell surface proteins, belongs to the tetraspanins family. From previous studies, we know that CD9 is considered to function primarily as a progression and metastasis suppressor in a variety of cancers, including breast, non-small cell lung colon and myeloma. However, an expanding body of literature has shown the contradictory outcome that tetraspanin CD9 is also vital in promoting cancer progression in several types of cancer. This review summarizes the recent studies on CD9 and concludes that it does not always act as a progression and metastasis suppressor. Conversely, in specific cases, CD9 may promote tumor progression through the following three aspects: Facilitating tumor cell transmigration, increasing tumor cell motility and hastening the growth of some cancers. In addition, CD9 appears to be an important marker of cancer stem cells in certain types of tumor.

## 1. Introduction

Tetraspanins are a family of integral proteins comprising 33 members, including the CD9, CD81, CD63 and CD151 proteins. They have been identified in mammalian cells and appear to be associated with a variety of cancer types (Table I) (1,2). Tetraspanins are membrane proteins containing a four-pass transmembrane domain, short N- and C-terminal cytoplasmic domains, a small intracellular loop, a small extracellular loop and a large extracellular loop (LEL). LELs may be subdivided into a constant region, which accounts for dimerization, and a variable region, which contains sites for interactions with non-tetraspanin partner molecules (1,3).

The tetraspanin CD9 gene is located on chromosome 12p13.3, coding a cell surface glycoprotein which belongs to the tetraspanin family and is expressed at a molecular mass of 25 and 28 kDa (4,5). CD9, also known as motility-related protein-1, was first identified as an antigen on lymphohematopoietic cells. Later, it was confirmed to be widely expressed in the plasma membrane of various normal cells, including smooth muscle, fibroblasts and several tumor cells (6–8). Similar to other tetraspanins, CD9 has been shown to be involved in a number of biological functions, such as cell adhesion, motility, cell migration, proliferation and differentiation (8–10). Numerous previous experimental studies and clinical results have demonstrated that CD9 functions as a progression and metastasis suppressor in several tumors. In addition, the downregulation of CD9 is tightly associated with a poor prognosis in breast cancer, non-small-cell lung cancer, colon cancer and multiple myeloma (11–14). The inactivation of CD9 may be due to epigenetic modifications, such as DNA methylation or histone deacetylation (14). A concept was gradually formed by previous studies that CD9 may selectively hamper several metastasis-promoting processes, including tumor cell motility, epithelial mesenchymal transition and recruitment of a protective environment (1). However, CD9 is not strictly classifiable as a metastasis and progression suppressor as several previous studies have confirmed that CD9, to some extent, contributes to the progression and metastasis of other types of malignances (15–18). Such a contradictory phenomenon reflects the complexity of CD9, which may vary with types of tumor and may also depend on different molecules in the tetraspanin web (5,19–21). The current review discusses the latent mechanism of CD9 in promoting the progression and metastasis of several malignancies.

## 2. CD9 may facilitate tumor cell transmigration

Previous examination of CD9 expression in certain cancers, including breast and colon cancers, has shown that metastases reduce CD9 levels compared with primary tumors (22,23). Nevertheless, an increasing number of studies have suggested that CD9 may be markedly associated with tumor endothelial cell (EC) interactions during transendothelial invasion. In breast cancer, differential gene profiling revealed that the expression of CD9 was higher in metastatic cancer cells when compared with that of the corresponding primary tumor cells, despite that in the majority of cases, expression of motility-related protein-1 (MRP-1/CD9) was downregulated in metastatic carcinoma cells (24). In addition, CD9 was significantly overexpressed in bone metastases versus primary tumors (25). In the light of a murine myeloma model conducted by De Bruyne *et al* who observed marked local expression of CD9 in myeloma cells in contact with bone marrow ECs, CD9 was found to possibly mediate homing and/or spreading of the breast cancer cells through interaction with ECs (15). In cervical cancer, CD9 expression is downregulated in the majority of invasive cervical carcinomas, but is locally re-expressed at the site of invasion into blood or lymphatic vessels. Sauer *et al* hypothesized that CD9 may be necessary for vascular dissemination during late tumor progression and existence of CD9 re-expressing cell clusters mediates the transendothelial invasion of cervical carcinoma cells. Therefore, CD9 may be used as an indicator of a high risk of recurrence in cervical cancer (17). In melanoma, CD9 re-expression was also observed in tumor vessels, suggesting that this protein is involved in tumor angiogenesis and in EC migration (26). It is supposed that CD9, expressed by ECs rather than melanoma cells, is localized at sites of contact between melanoma and ECs. These reports also showed that ECs exhibit an active redistribution of CD9 to points of tumor cell insertion during the extravasation phase of tumor metastasis (27).

The mechanism of the abovementioned role of CD9 is unknown. Klein-Soyer *et al* highlighted the important role of CD9 in EC migration during lesion repair angiogenesis through cooperation with integrins (28). Recent studies have provided evidence that CD9 hot spots between cells and ECs support not only angiogenesis through *in vitro* EC migration and invasion induced by vascular endothelial growth factor (VEGF) or hepatocyte growth factor (HGF), but also lymphangiogenesis by interacting with VEGF receptor 3 and integrins in human lymphatic ECs (29,30). Prior to the stage of the CD9 mediating interactions between tumor cells and ECs, matrix metalloproteinases (MMPs) are important in tumor invasion and metastasis. In human melanoma, the CD9 tetraspanin protein appears to contribute to the transendothelial invasion of cancer cells by stimulating MMP-2, which degrades the extracellular matrix (ECM) surrounding ECs (31). In addition to MMP-2, membrane type-1 MMP (MT1-MMP), known as a proinvasive protease during tumor cell invasion, also coimmunoprecipitates and colocalizes with CD9 (32). In addition, invasion and metastasis may be elevated through preventing lysosomal degradation of MT1-MMP by CD9 in cancer cells (33).

In conclusion, we speculate that specific types of tumor expressing high levels of CD9 destroy the ECM through triggering the activity of MMPs, allowing them to contact with vascular ECs. The tumor cells are influenced by cytokines, such as VEGF or HGF, and transmigrate easily through ECs into the circulation.

## 3. CD9 may enhance tumor cell motility

In the early 1990s, Ikeyama *et al* introduced a human MRP-1/CD9 complementary DNA plasmid into several cancer cell lines and found the suppression of cell motility following transfection, suggesting that CD9 renders tumor cells more static *in vivo*, consequently resulting in the suppression of tumor metastasis (8). However, to date, the manner in which CD9 inhibits tumor cell motility remains unclear. Possibly, CD9 hinders metastasis formation by prohibiting integrin-mediated motility of cancer cell lines from lung, breast, skin, gastric, pancreatic and bladder tumors *in vitro* (34). Other studies have considered that CD9 may downregulate the expression of Wiskott-Aldrich syndrome protein 2 (WAVE2) and that the decreasing WAVE2 then affects the actin cytoskeleton and suppresses tumor cell motility (35,36). Paradoxically, it is increasingly appreciated that CD9 also provides cells with powerful motility in several carcinomas.

Besides promoting metastasis through interaction with ECs, melanoma cells with high levels of CD9 become more motile. In order to understand the expression and function of CD9 in melanoma cells, Fan *et al* performed an investigation in non-malignant mouse melanocytes, normal human cutaneous melanocytes, B16 mouse melanoma and specific human melanoma cell lines. The authors found that, although CD9 expression is reduced in the transition from melanocytes to melanoma, the re-expression of CD9 in specific melanoma cells alters the microenvironment of the tumor and leads to the enhanced invasion to Matrigel (16).

Detailed studies analyzing CD9 have previously indicated that CD9 alone does not inhibit tumor cell motility. Ganglioside GM3, an important lipid composition of the plasma membrane, cooperatively inhibits haptotactic tumor cell motility through interacting with CD9 (37). However, it has been demonstrated that the motility of tumor cells essentially depends on the expression of GM3. High levels of GM3 expression interacting with high levels of CD9 cooperatively reduces tumor cell motility by inhibiting c-Src activation. Conversely, low GM3 levels but high CD9 levels enhance tumor cell motility through activating c-Src (38). This is why specific tumor cells, expressing higher levels of CD9, are more motile.

CD9 associates with a variety of β1 integrins (39). Consistent with their previous studies, revealing that ectopic expression of CD9 in a Chinese hamster ovary cell model system causes increased haptotactic motility to ECM proteins (such as fibronectin), Kotha *et al* demonstrated that CD9, in concert with integrin α5β1, promotes cell motility via a phosphatidylinositol-3 kinase-dependent pathway (40,41). Several lines of evidence have indicated that integrins are important regulators of cell motility and their dysregulation may be associated with tumor progression (41,42). Pellinen *et al* identified with loss-of-function and rescue experiments that CD9 activates β1 integrins, which are positively linked with cell motility, such as invasion, in prostate cancer cells (43). Similarly, CD9 also associates with numerous other membrane proteins, such as immunoglobulin protein, in particular microdomains on the plasma membrane. CD9P-1 is a cell surface protein with immunoglobulin domains specifically interacting with CD9. In pathological conditions, such as cancer, the expression levels of CD9 may induce marked effects on cell motility through a change in the membrane compartmentalization of CD9P-1, thus favoring metastasis. Therefore, it has previously been hypothesized that the ratio of expression levels between CD9P-1 and its tetraspanin partners collectively modulates cell motility (44).

CD9 not only promotes the development of bone metastases of MDA-MB-231 breast cancer cells, as previously described, but also enhances the motility of this cell line. Notably, native type IV collagen induces a transient increase of CD9 levels on the surface of MDA-MB-231 breast cancer cells and then increases their motility, such as invasion, through interaction with CD9 (45).

Consequently, we propose that whether CD9 increases the motility or invasion of tumor cells depends on the prevailing conditions, particularly the partner proteins associating with CD9.

## 4. CD9 contributes to the proliferation and survival of tumor cells

A number of previous studies have analyzed the effect of the tetraspanin, CD9, on the life of tumor cells in several types of cancer. For example, Murayama *et al* described that CD9-mediated apoptosis in human cells is through triggering the activation of c-Jun N-terminal kinase/stress-activated protein kinase and p38 mitogen-activated protein kinases, as well as caspase-3 (46). Zvereff *et al* elucidated that CD9 interaction with mortalin causes cell death via mitotic catastrophe in prostate cancer cells, showing the importance of CD9 in tumor suppression (47). In addition, Shallal *et al* found that CD9-transfected myeloma cell lines are more susceptible than control-transfected or non-transfected parental cells to cell-mediated lysis by immune cells (48). By contrast, in specific cases, CD9 appears to enable tumor cells to grow and proliferate more vigorously.

Previously, it has been recognized that heparin-binding epidermal growth factor-like growth factor (HB-EGF) which interacts with CD9 is a membrane-binding cell proliferation factor of the epidermal growth factor (EGF) family and is involved in tumorigenesis and the proliferation of human gastric cancer. A previous study conducted by Murayama *et al* showed that HB-EGF mRNA levels were higher in gastric cancer than in normal gastric tissues. Subsequently, Hori *et al* speculated that CD9 may accelerate the proliferation of gastric cancer cells via interaction with HB-EGF on cell membranes (18,49). A similar phenomenon is also observed in multiple myeloma, that CD9 may increase tumor proliferation through increasing the ability of the HB-EGF/EGF receptor and CD9 may contribute to the proliferation of tumor cells by associating with HB-EGF on cell membranes (50). In addition, myeloma cells with higher levels of CD9 expression proliferate rapidly via interleukin (IL)-16. Since CD9 was reported as an IL-16 receptor, Atanackovic *et al* proposed that the myeloma cell proliferation may be triggered by IL-16 through interacting with CD9. The authors also suggested that CD9 be further evaluated as a possible therapeutic target for multiple myeloma in the future (51,52).

Conflicting data exist with regard to CD9 in ovarian cancer. A recent study produced a contradictory result that revealed CD9 to be upregulated in ovarian carcinomas, particularly in serous-type ovarian cancer. In addition, CD9 may function as an antiapoptotic protein through inducing cytokines, such as tumor necrosis factor α, IL-6 and IL-8, as well as constitutively activating the nuclear factor-κB signaling pathway (53).

Tetraspanin CD9 may prevent cancer cells from being killed by chemotherapeutic agents. More recently, Kohmo *et al* revealed that CD9 is highly re-expressed in small cell lung cancer cells (SCLCs) at relapsed primary tumors and metastasized organs in patients who have received chemotherapy. These recurrent SCLCs appear to be chemoresistant through a cell adhesion-mediated drug resistance mechanism. In addition, selective inhibition of CD9 inducing the apoptosis of chemoresistant SCLC cells implicated that CD9 may serve as a protective factor for SCLCs in ultimately resistant stages (54).

## 5. CD9 and cancer stem cells (CSCs)

It has been previously reported that tumors may contain CSCs, defined as a small subpopulation exhibiting self-renewal and differentiation abilities. The origin of CSCs is indistinct and some reports have suggested that CSCs may stem from normal tissue stem cells (55). Thereby, CSCs own similar biological characteristics with normal stem cells and have the potential of initiating tumor development, leading to metastasis and even recurrence (55,56).

In normal stem cells*,* such as mesenchymal stem cells (MSCs), CD9 is important in increasing the MSC proliferation, which has been confirmed by Kim *et al* (57). In human testicular germ cell tumors, particularly in intratubular germ cell neoplasia unclassified (IGCNU), CD9 has been deemed a core marker of pluripotent embryonic stem cells and is upregulated. This suggests that CD9 has an underlying impact on the expression of stem cell genes and maintenance of the undifferentiated state in IGCNU (58). Previously, Nishida *et al* performed a comprehensive analysis of surface markers on several B-ALL cell lines and identified that CD9 is a useful positive-selection marker for the identification of CSCs in human B-acute lymphoblastic leukemia and may also serve as a novel therapeutic target in this disease (59). For the sake of achieving a deeper insight into the CSC properties in CD9-expressing cells of B-ALL cell lines, the authors performed detailed assays and showed that CD9 regulates the cancer-related genes, such as TEL/AML1 and E2A/PBX1, in B-ALL and significantly affects Src family proteins involved in diverse biological functions. Furthermore, CD9^+^ cells of B-ALL exhibited drug-resistance (60). Notably, CD9, as a cancer stem cell marker, may also be applied to human malignant mesothelioma (61).

## 6. Conclusion

From the abovementioned findings, we conclude that tetraspanin CD9 does not always act as a tumor suppressor. In certain types of carcinoma, CD9 propels tumor progression and metastasis. The underlying mechanisms may involve the following aspects: i) The interaction between CD9^+^ cancer cells and ECs is important in transendothelial migration of the tumor cells; ii) CD9 is likely to enhance the motility, such as invasion, in several types of cancer; iii) CD9 promotes tumor cell growth and prevents apoptosis from chemotherapy drugs; and iv) CD9 is becoming an increasingly important indicator for the identification of CSCs, since CSCs are crucial in oncogenesis. Future studies are required to appreciate the manner in which CD9 contributes to the cancer phenotype.

## Figures and Tables

**Table I:** Expression of tetraspanins in normal tissues.

Tetraspanin	Common name	Expression	Chromosome
CD9	MRP-1	Endothelial cells, vascular smooth muscle cells, platelets and myeloid cells	12p13.3
CD82	Kangai1	Prostate, lung, liver, kidney and bone marrow	11p11.2
CD151	PETA3	Epithelial and endothelial cells	11p15.5
CD63	MEL1	Lysosome	12q12–q13
Tetraspanin 8	CO-029	Capillary endothelial cells, nerves and smooth and striated muscle cells	12q14.1–q21.1

MRP-1, motility-related protein-1.

## Abbreviations

MRP-1: motility-related protein-1
CSC: cancer stem cell
LEL: large extracellular loop
VEGF: vascular endothelial growth factor
HGF: hepatocyte growth factor
MMPs: metalloproteinases
ECM: extracellular matrix
WAVE2: Wiskott-Aldrich syndrome protein 2
HB-EGF: heparin-binding epidermal growth factor-like growth factor
SCLC: small cell lung cancer cell
MSC: mesenchymal stem cell
IGCNU: intratubular germ cell neoplasia unclassified
